# Using social media listening to identify the real-world challenges faced by dog owners globally when administering oral medications

**DOI:** 10.3389/fvets.2025.1502236

**Published:** 2025-06-30

**Authors:** Georgina Tarrant, Taranpreet Rai, Andrea Wright, Travis Street, Kevin Wells

**Affiliations:** ^1^Surrey DataHub, vHive, School of Veterinary Medicine, University of Surrey, Guildford, United Kingdom; ^2^Centre for Vision, Speech and Signal Processing, University of Surrey, Guildford, United Kingdom; ^3^Outcomes Research, Zoetis Inc., Parsippany, NJ, United States

**Keywords:** social media, large language models, pulsar platform, topic modeling, palatability, oral formulations, canines, dogs

## Abstract

**Introduction:**

Pet owner compliance with oral medication administration represents a significant challenge in veterinary medicine, yet limited real-world data exists on the experiences and barriers faced by dog owners during at-home “pilling” processes.

**Methods:**

This study employed social media listening (SML) to analyze 4,787 relevant posts from X, Reddit, Facebook, blogs, and forums discussing oral medication administration to dogs. Large language models (LLMs) were used for data cleaning, relevancy filtering and analysis. Topic modeling, sentiment and thematic analyses were conducted to identify key themes and challenges.

**Results:**

Analysis revealed significant anxiety and fear associated with medication administration, with 12% of posts mentioning anxiety and additional fear-related terms appearing in 1-3% of posts. Only 7.6% of posts discussed soft/chewable medications, which showed positive sentiment and preference. Geographic analysis showed posts predominantly from English-speaking countries (US 70.3%, UK 17.8%). Five major themes emerged from X, Reddit, blogs and forums: general medication/veterinary experiences, pill types and practices, pill delivery methods, specific medications/preventatives, product reviews/natural remedies. Financial concerns were prominent, with pet owners describing medication costs as barriers to optimal care. Successful pilling strategies included hiding pills in peanut butter, cheese or meat products; crushing pills and mixing with food, and using distraction techniques.

**Discussion:**

The study identified key barriers to compliance including financial constraints, fear and anxiety, mistrust of veterinary advice and practical administration challenges. Pet owners showed higher adherence when treatments visibly improved quality of life or addressed chronic conditions. Chewable formulations were preferred but raised concerns about accidental overdosing. The methodology demonstrated that SML combined with AI analysis effectively captures real-world pet owner experiences.

**Conclusions:**

This novel approach revealed that dog owners face significant psychological and practical barriers when administering oral medications. Chewable formulations may improve compliance, though proper storage and education are essential. The study provides veterinarians with evidence-based successful pilling strategies reported by pet owners and highlights the need for better communication about treatment benefits, financial planning options and alternative delivery methods to improve medication adherence.

## 1 Introduction

Medication adherence in dogs, particularly for oral dosing, is crucial for the effective management of various health conditions. Common ailments requiring oral medications include infections, skin disease, short-term pain (e.g., post-operative), chronic pain from diseases like arthritis, allergic dermatitis, and controlling parasitic infestations (1, 2). However, few studies have explored factors affecting compliance with medication regimens (3, 4). Such studies have typically shown that pet owner adherence to veterinary recommendations for oral medications falls short of ideal coverage (5). For example, Lavan et al. found that while almost all veterinary hospitals in their study recommended 12 months of flea and tick prevention, the estimated actual coverage was only 6.1 months based on owner medication purchases from their veterinary hospital over a 12-month period (6). This gap between recommendation and practice is further highlighted in a separate study by Lavan et al., which revealed that a considerable proportion of dog owners purchased only one or two doses of oral flea and tick medication per year. Specifically, 30 to 42% of dog owners obtained only one dose of oral flea and tick medication per 12-month period, depending on the product (7). A study by Mwacalimba et al. showed a compliance rate of 7.1 months for monthly heartworm preventatives in Australia, while a similar study by Mwacalimba et al. showed compliance of 7.3 months in the United States (8, 9). A further study by Mwacalimba et al. found that 25% of pet owners in the United States purchased a heartworm preventative at least once in a 2-year period (10).

Purchase compliance for chronic conditions and regular treatments among dog owners is generally thought to be low (11), which concomitantly impacts outcomes. Pet owners’ compliance with preventative medicines is particularly challenging as owners may question antiparasitic treatment necessity, perceive veterinarians as “upselling” products, or be concerned about side effects (12). Similarly, suboptimal compliance with other medications may lead to suboptimal treatment outcomes, prolonged recovery times, and drug resistance development. Veterinary practice management strategies such as monthly payment plans and creating tailored annual care plans for pets can help to address financial barriers to treatment (13, 14).

Pet owners’ dosing adherence to routine daily, weekly, or monthly long-term veterinary prescription recommendations is crucial for treatment efficacy. Maintaining consistent medication schedules for dogs can be challenging, particularly for long-term treatments (e.g., parasite prevention and arthritis pain medication) and chronic conditions requiring daily administration (e.g., allergic dermatitis). Other challenges include treatment duration, inability to give medicine with food, inconvenient dosing schedules, administering multiple prescribed medications, perceived adverse effects causing pet owners to stop administering medication, and complex treatment plans (3, 15–17). Some owners discontinued medication after observing possible side effects such as diarrhea, while many modified medications without veterinary advice by opening capsules, crushing tablets, or changing dosing schedules to facilitate administration (3). Compliance improves when veterinarians explain medications, simplify the treatment regime, and demonstrate how to administer the medication (3). Communication is vital to determine an owner’s priorities, and treatment should be customized to both patient and owner. Recent studies indicate that longer-acting medications may improve compliance in areas such as ectoparasite protection (6, 11, 18–20). However, prolonged drug use can reduce efficacy. For example, extended, repeated, or poorly managed antibiotic use in dogs can contribute to antimicrobial resistance (AMR), especially for chronic conditions requiring extended therapy, such as recurrent skin infections, urinary tract infections, or osteomyelitis (21). It has been found that long-term exposure to monthly fipronil treatments has selected for resistant flea strains (22), and resistant Dirofilaria immitis strains that cause heartworm disease have emerged (23).

Successfully administering regular oral medications to dogs is thought to represent a momentous challenge for many pet owners (1, 3, 5, 17, 24), with approximately one-third of pet owners in a New Zealand study reporting difficulties administering prescribed medications to their pets. Pet owners reported several strategies that helped with medication administration, including using food, modifying their behavior or their pets, using positive reinforcement, or combining multiple approaches (3). Canine resistance to administration of oral medications is thought to be the most common challenge (3). This often stems from factors such as a perceived unpleasant taste, smell, or associated anxiety. Aleo et al. found that sophisticated flavor enhancers that improve taste, aroma, and texture can increase a dog’s willingness to consume tablets (25). As a result, various formulations have been developed, including film-coated tablets, capsules, powders, liquid solutions, and chewable and palatable tablets designed to improve treatment adherence. However, even with flavor enhancers, excessively chewy tablets may still be refused. Adding an enticing scent can encourage dogs to pick up a tablet (1, 5). Chewable formulations are considered a palatable alternative to film-coated tablets and have been shown to increase compliance through ease of administration and increased acceptance by dogs (26). Various strategies can be employed by pet owners to encourage ingestion, although these approaches may not always be successful. Soft gelatin capsules can improve dosing compliance, particularly if the capsule size is decreased to make it easier for the dog to ingest. This suggests that optimizing drug delivery systems to make the pills more palatable is likely to enhance dosing compliance (27).

This study investigates the practical challenges pet owners face when administering oral medications to dogs, comparing different types, such as coated pills, soft chewables, and flavored or flavorless pills. Prior work has relied on surveys and/or focus groups and may exhibit sensitivity to participant selection bias and response bias to questions. A complementary alternative is to employ social media listening (SML) data with AI-driven (artificial intelligence-driven) topic modeling and regional demographic analysis. Using this approach, we examine ‘the collective wisdom of the [online] crowd’ by extracting unsolicited real-world pet owner experiences publicly shared on social media (SM) platforms. Key themes are identified using AI tools and correlated with demographic data to provide an enhanced understanding of canine medication administration issues in pets across the globe.

Social media listening is a powerful tool for extracting insights quickly and efficiently from text content (28). SML involves actively tracking and analyzing unsolicited online content related to specific topics across SM media to understand sentiment, trends, and behavior (29). This technique can be used to explore a variety of topics from pet owner perceptions of feline and canine pruritus (30–32) to the role of women in livestock farming in Sub-Saharan Africa (28). In this study, we harness SML and AI topic modeling to understand the challenges experienced by pet owners around the world when administering oral medications to their dogs.^1^

The aims of this study were to (i) better understand practical and commonly encountered challenges experienced by pet owners when administering oral medications to dogs of all sizes, (ii) to examine the unique challenges of medicating large dogs, and (iii) to examine the experience of different pilling types and administration methods.

## 2 Methodology

**Figure 1:**
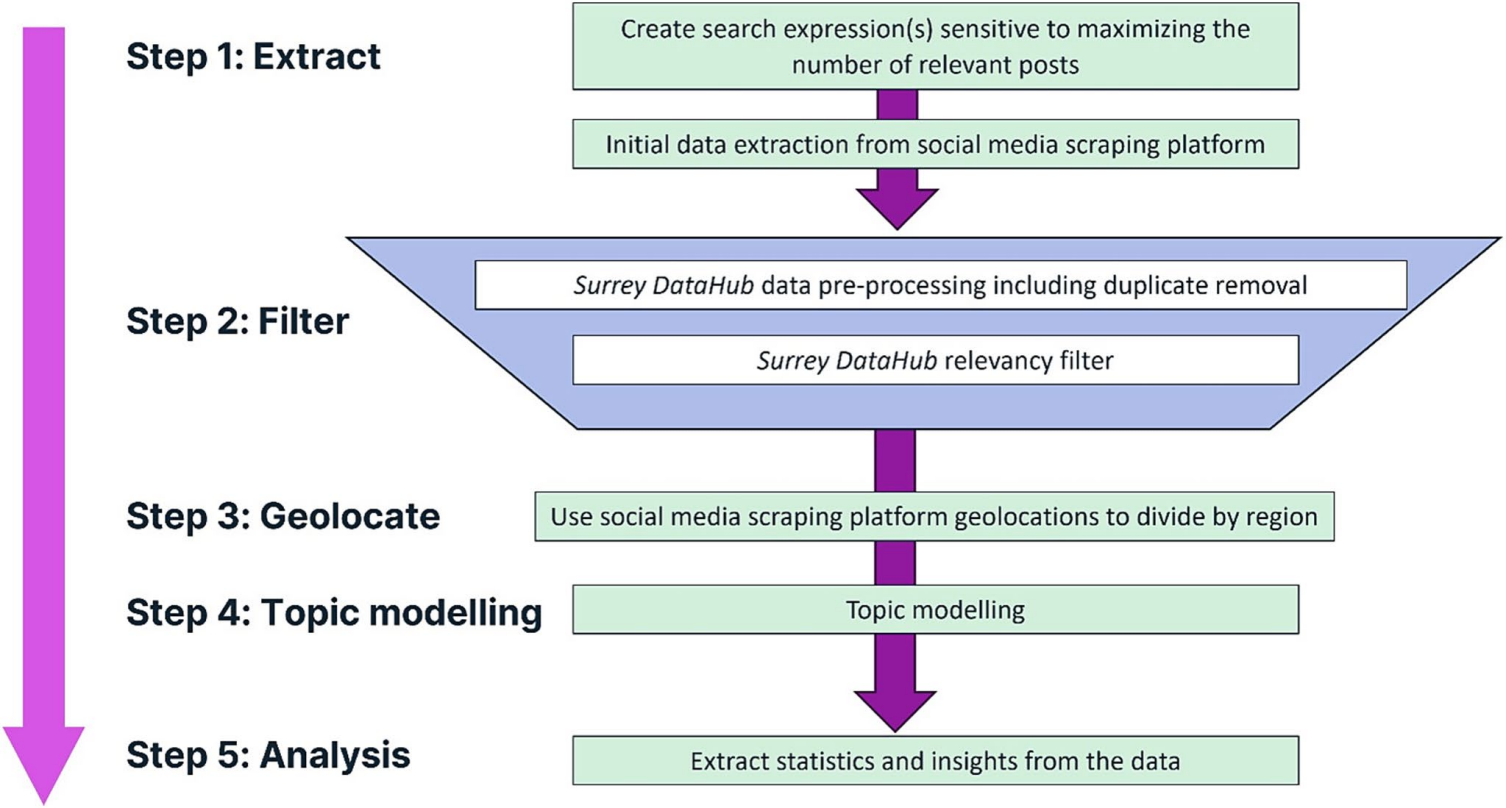
Overview of the data collection and analysis process.

An overview of the data collection and analysis workflow is provided in Figure 1. In brief, we use a commercial SML platform (designed for marketing applications such as tracking brand awareness) to ‘scrape’ SM content. This process is initiated by creating tailored search expressions to collect SML data. Data are then cleaned and pre-processed (to remove advertisements/promotions, irrelevant content, retweets/reposts, etc.) and filtered to extract key SM posts for downstream analysis.

### 2.1 Data collection

**Figure 2:**
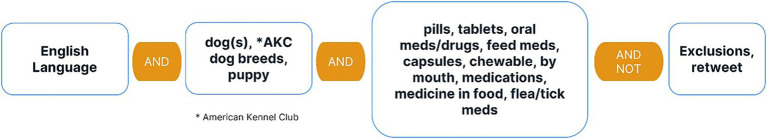
Boolean expression developed to scrape social media content from X, Reddit, blogs, and forums using Pulsar Platform™.

To extract (commonly referred to as ‘scrape’) SM data, either bespoke software can be employed (that may have legal data implications) or a variety of subscription-based commercial SML platforms may be used, e.g., Sprout, Hootsuite, Meltwater, etc. These have large-scale scraping agreements and licenses with major SM outlets such as X (formerly Twitter), Reddit, Instagram, etc. We have used Pulsar Platform™, one of the largest and best-known SML platforms, to conduct topic searches using bespoke keywords and key phrases. A tailored set of search expressions was developed to maximize sensitivity for retrieving SM posts relevant to administering oral medications to dogs of all sizes, particularly daily pilling with chewable, flavored, or non-flavored products, in conjunction with expert veterinary information. The structure of the Boolean logic search expression is shown in Figure 2. The search was limited to anglophone posts worldwide. American Kennel Club (AKC) dog breeds were used to expand the search beyond mentions of dogs. Retweets were excluded as these are a form of duplicate in this context. Other exclusions were added as necessary. Further details on Facebook scrapes are available in Supplementary Table S1.

Pulsar Platform™ was used to scrape SM posts from X, Reddit, blogs, and forums. A time window of 30 days was selected for the data scraping from all sources. X, Reddit, blogs, and forums were scraped from 4th May to 10th June 2024. A maximum of 30 days of historical data are available from Facebook; therefore, this platform was scraped from 7th May to 6th June 2024.

Preliminary raw data were manually reviewed after 24 h, and revisions were made to the search exclusions to improve the quality of the resulting data. Bulk scraping of SM frequently produces irrelevant data despite careful optimization of search terms. As such, the analysis of raw SM data from any platform needs to be treated with caution. This is often due to the coincidental use of keywords on irrelevant topics or posts that are impossible to anticipate. In this study, we inadvertently scraped a large number of derogatory, racist, and/or misogynist posts, as this coincided with the derogatory/emotive use of words pertinent to this topic. Adult/pornographic content was similarly problematic. This and related terms were excluded in so far as possible via exploratory pilot work before the initial scraping of the raw SML data, and further exclusion processes were needed at the pre-processing stage to avoid contaminating the SML data. This kind of contamination can routinely account for a substantial amount of SML data and highlights the importance of cleaning and filtering SML data before conducting any substantial analyses.

Results (SM posts) from all 33 Pulsar searches were downloaded when the 30-day search was complete, resulting in ~27 k SM posts.

### 2.2 Data cleaning and pre-processing

It was necessary to clean and pre-process the data before filtering for relevancy and conducting analyses.

SML data often contains duplicates across different scrapes. These duplicates arise for various reasons. One cause was overlaps between searches created manually. For example, a broad and potentially noisy search for “medications” might be followed by a narrower search for “oral medications.” This is done to examine whether the narrower search has sufficient coverage. Another source of duplicates was posts that are retweets but miss the retweet exclusion. This can happen due to tagging issues on X. Duplicates can also occur when posts are published by the same user on multiple SM platforms. Reposts by the same user on the same forum can also create duplicates. This might happen if the original response was not as the user desired.

The X, Reddit, blogs, and forums data were cleaned to remove duplicates using Python, and an LLM (GPT-4) was used to repeat this process for the concatenated Facebook data. We decided not to remove special characters (e.g., emojis) from the data at this stage as these might provide additional context to the posts. The title and post content were concatenated to optimize zero-shot relevancy filtering by providing context relating to the data source.

After filtering, 75% of the posts were retained, ensuring cost-effective and efficient analysis. Posts exceeding 640 words were then removed to minimize the complexity and costs associated with processing through LLMs, as longer posts often contribute less relevant content. Manual spot checks of posts longer than 640 words were implemented, confirming this observation. Additionally, posts with fewer than 15 words were excluded to enhance the accuracy of zero-shot classification, as these shorter entries typically lack sufficient context for reliable classification. See Supplementary Figure S1 for a histogram justifying this word limit.

### 2.3 Zero-shot relevancy filtering

In one of our previous SML studies (33), data scraped from publicly available SM was manually labeled as relevant or irrelevant to the topic under investigation by a human reviewer: a tedious and potentially error-prone task. By contrast, in this latest work, we leveraged an LLM (OpenAI’s GPT-4 model) for zero-shot classification through “prompt engineering,” an iterative process where users provide conversational instructions and examples to the LLM to optimize performance (28, 34). Thus, an LLM categorized SML posts, with a representative sample of classified posts verified by a human. Our test runs demonstrated that GPT-4 was more accurate in classifying the relevancy of the post than ChatGPT. A sample of the posts labeled as ambivalent was manually spot-checked, and it was found that most of these were irrelevant (advertisements or similar promotions).

### 2.4 Audience profiling, demographic, geographical, and sentiment analysis

Once filtered for relevancy, a total of 3,629 relevant posts from X, Reddit, blogs, and forums and a further 1,158 relevant posts from Facebook were used for subsequent analysis out of a total of 26,846 posts contained in the original raw data.

Sentiment analysis was applied to these filtered posts using the Pulsar Platform™ sentiment AI model (35). Posts were automatically assigned sentiment scores of −1, 0, or 1, indicating negative, neutral, or positive attitudes. The sentiment model recognizes explicitly positive/negative words and their context, understanding negations such as “not good” and modifiers such as “really good.” Geographical analysis of the individuals posting by country and city was undertaken in some cases. X platform has sophisticated geolocation capabilities for many users, while Facebook is more limited, as posts within a group are assigned to the location of the group or the page owner. The authors of posts on Reddit, blogs, and forums can be geolocated using the country code of the internet domain or the author’s location where available.

A key aim of the study was to investigate the challenges faced by pet owners when administering pills to large dogs specifically. To this end, we specifically further segmented data for large dog breeds by considering explicit mentions of large dogs or synonyms for large dogs, mentions of breeds with a “standard” size, and 26 AKC large breeds along with their synonyms. A further four filters were developed to detect mentions of different oral medication types, specifically pills that are (i) shiny or coated, (ii) flavored, (iii) non-flavored, and (iv) soft chewables.

### 2.5 Topic modeling

Data scraped from X, Reddit, blogs, and forums were analyzed first. The topic modeling themes were generated by combining Non-Negative Matrix Factorization (NMF) and an LLM, in this case, GPT-4o, to identify key topics within the data. NMF is a well-known topic modeling method (36) and the goal is to identify word/topic patterns or structures within the data (28, 37). The LLM was prompted to interrogate the data and extract topics. From the top five topics, the LLM was prompted to output the top 20 keywords associated with each topic for manual checking.

The next stage of the topic modeling analysis involved making sense of the ‘word salads’ that arose from the above step. Conventionally, this can be undertaken manually; however, we chose to explore an automated approach that used prompting with an LLM to make sense of the data-driven topics and generate plausible themes that could be understood by a human observer instead. The LLM was prompted to generate a description of each topic and assign individual SM posts to the topics. For data quality checks, the LLM was also required to provide example posts matching each theme, and these were easily manually checked against the input data. All extracted exemplar posts from each theme existed within the raw data, although some of the examples were slightly re-worded by the LLM. This process was repeated for Facebook data.

Following the initial topic modeling exercise, the data were further interrogated with specific probes to retrieve any posts discussing specific issues associated with pilling.

## 3 Results

### 3.1 Data collection, processing, and filtering

**Figure 3:**
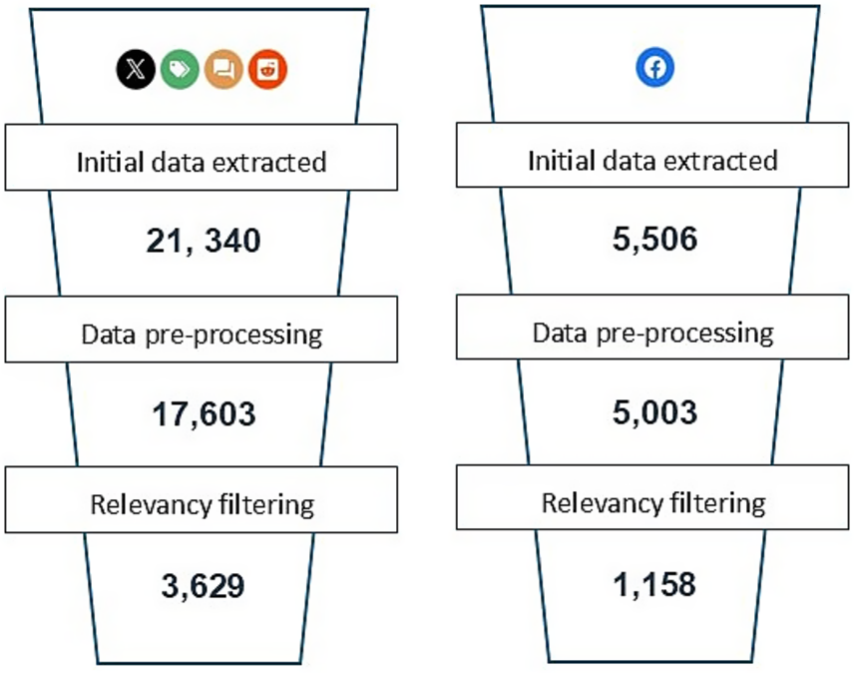
Pipelines showing total social media posts extracted from X, Reddit, blogs, forums, and Facebook (raw data) through the data cleaning and pre-processing stage and zero-shot relevancy filtering. The raw data from Facebook were exported from 22 separate Pulsar Platform™ searches and concatenated.

The initial data extraction from Reddit, X, blogs, and forums using Pulsar Platform™ resulted in a raw dataset containing 21,340 posts, as shown in the data funnels illustrated in Figure 3. Following data pre-processing to remove blank posts (e.g., Reddit posts that had been deleted after publication, leaving the title available but devoid of content), duplicates, and posts <15 words or >640 words, 17,603 posts were remaining. These posts were filtered using a zero-shot approach utilizing the GPT-4 LLM as described previously. This left a total of 3,629 posts for analysis.

A total of 5,506 Facebook posts were scraped using the 32 Facebook searches on Pulsar Platform™. Only 22 of the 32 Facebook searches contained relevant data; the remaining 10 Facebook searches found zero posts in the selected period. Zero duplicates were found, and ~90% of posts remained after removing posts <15 words and >640 words. The pre-processing step removed 503 posts to leave 5,003 posts. The processed posts were filtered using the GPT-4 zero-shot relevancy filter, leaving 1,158 posts remaining for analysis, as shown in Figure 3.

### 3.2 Audience profiling and demographic analysis

Only ~16% of posts could be confidently predicted as male or female, with most posts by users of unknown gender. There were 3,201 unique authors in the dataset, of which 8.4% (*n* = 272) were female and 8.1% (*n* = 262) were male. The remaining authors were of unknown gender (*n* = 2,667).

However, keywords in user biographies on X platform, the only platform scraped that provides this information, appeared to show a slant toward female posters, with “mom,” “children,” and “family” being more closely associated with female posters. Slightly more male than female posters were observed for the large dog breeds, but the sample size was considered to be too small to draw meaningful conclusions. Studies have shown that men often own larger, sporty dog breeds, as this reflects the owner’s self-image (38).

According to the available demographic information from author’s user biographies on the X platform, the most frequently mentioned interests were “dogs” (*n* = 34, 29.1%) and “mom” (*n* = 32, 27.4%), the pronouns “she/her” (*n* = 31, 26.5%) and “animal” (*n* = 20, 17.1%). Not all X users have an author biography; therefore, these data are not available for some authors.

**Figure 4:**
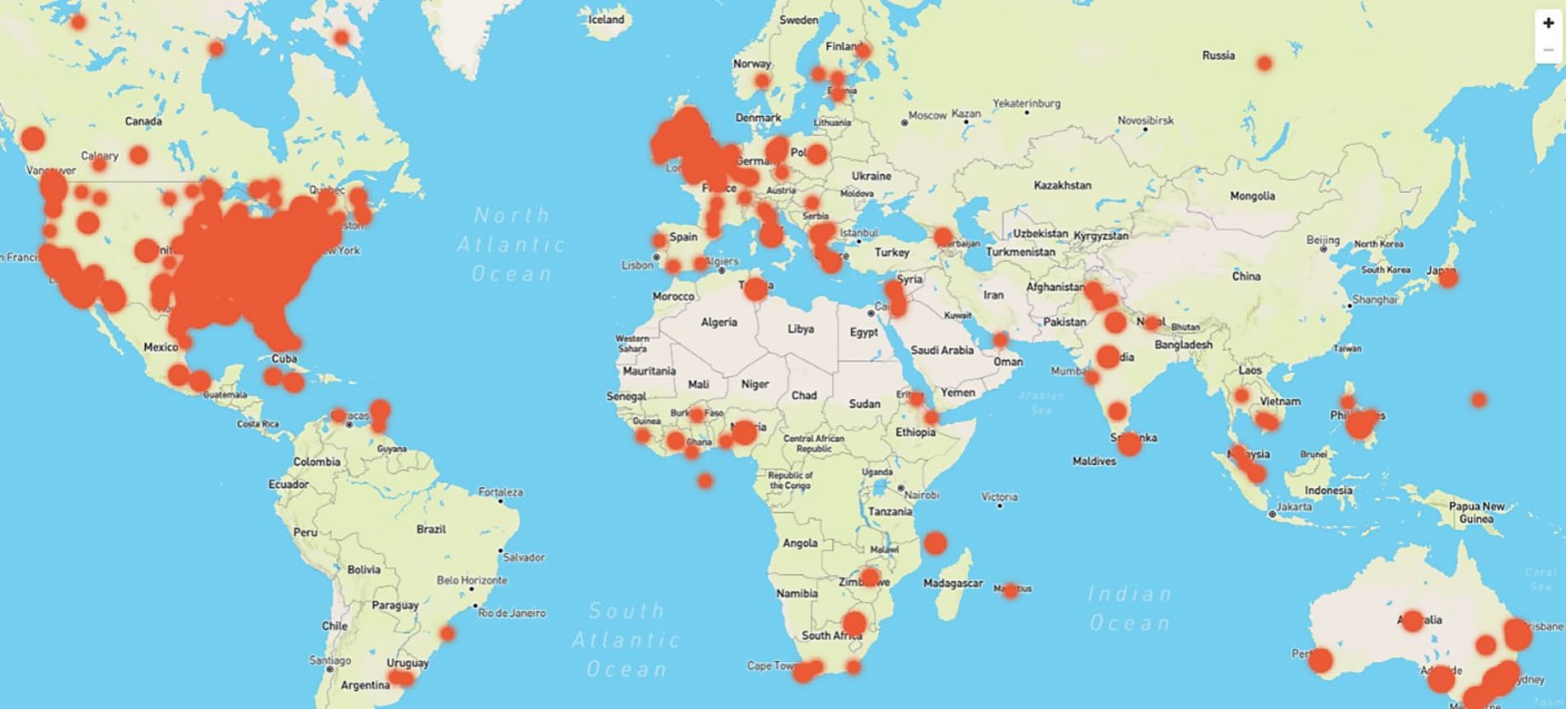
Coordinates map showing where the majority of social media posts discussing the administration of oral medications to dogs originate from, broken down by geo-coordinates.

The majority of posts (see Table 1 for the distribution of posts between the top four countries) were authored by people from the United States (*n* = 445, 70.3%), with other English-speaking countries represented: United Kingdom (*n* = 144, 17.8%), Australia (*n* = 81, 7%), and Canada (*n* = 44, 5%). Location data are deduced from the authors’ profile or geolocation data. The posts collected were largely from anglophone nations, and the search expressions were written in the English language. Posts were collected from numerous countries in addition to the top four countries included in Table 1. Geolocation of social media posts discussing the administration of oral medications to dogs by the top four countries with a sentiment (positive, negative, and neutral). Figure 4 demonstrates the full geographical spread of post locations. This shows that the SML narrative around dog pilling had global reach despite the dataset being limited to posts in English rather than other languages. The geolocations of the posts are focused on cities, although this could simply reflect the larger human and dog populations in these areas.

**Table 1:** Geolocation of social media posts discussing the administration of oral medications to dogs by top four countries with sentiment (positive, negative, and neutral).

Countries	Results (posts)	Positive	Negative	Neutral
United States	445	258	176	11
United Kingdom	144	64	39	41
Australia	81	42	13	26
Canada	44	17	15	12

### 3.3 Sentiment analysis

**Figure 5:**
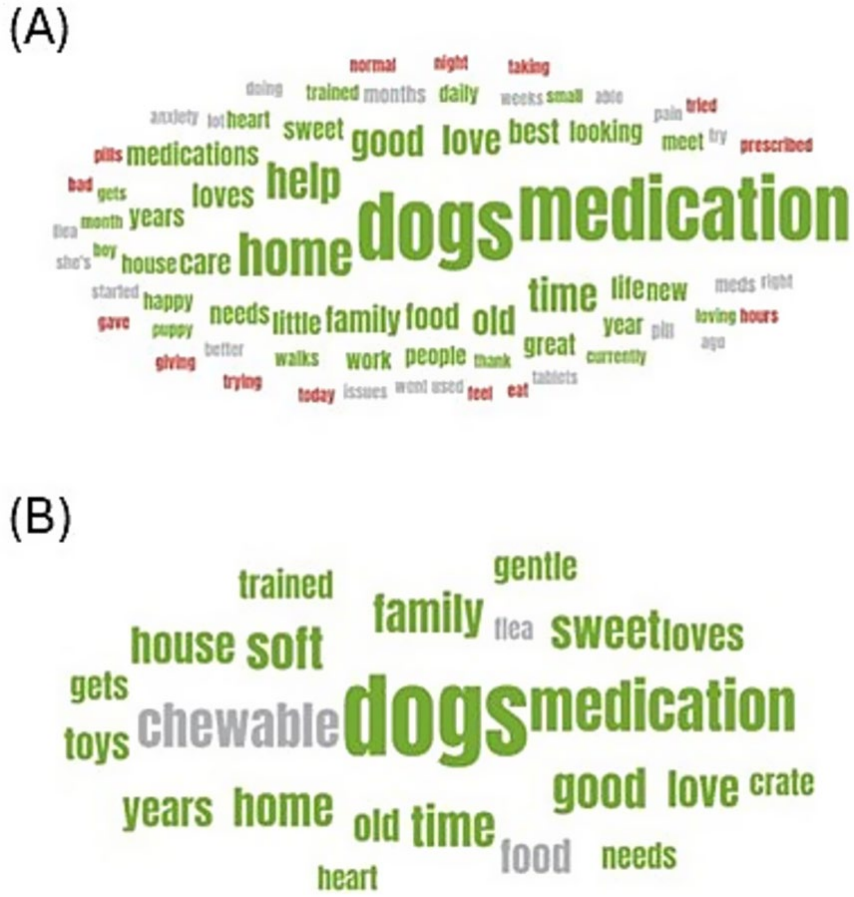
Wordcloud **(A)** shows the most frequently mentioned keywords in social media posts relating to pet owner experiences administering oral medications to dogs by sentiment, and **(B)** shows the most frequently mentioned keywords in social media posts relating to pet owner experiences administering chewable oral medications to dogs by sentiment. Green is a positive sentiment, red is negative, and gray is neutral. Keyword size is proportional to the frequency of mentions of the keyword.

The relevant SM posts were analyzed by sentiment and labeled positive, negative, or neutral within the platform. The wordclouds in Figure 5 show the keywords from the posts by relative word frequency and sentiment for (A) oral medication administration and (B) chewable medication administration. A wordcloud is a visual representation of text data, where the size of each word corresponds to its frequency within the corpus of text, and the color of the keyword indicates the sentiment attributed to it. The majority of keywords had a positive sentiment, particularly in relation to “medication,” “home,” and “help.” It is interesting to note that mentions of “pain,” “pill,” and “tablets” had neutral sentiments, while mentions of “pills,” “bad,” “eat,” “prescribed,” “trying,” “gave,” and “giving” were negative.

### 3.4 Pill-type content within social media data

The relevant SM posts were filtered to find posts that mention coated pills, flavored (palatable) pills, unflavored pills (including flavorless, odorless, and plain), soft or chewy medications, and posts discussing the challenges of administering pills to large dogs based on mentions of large dogs and AKC large dog breeds. Additional oral medications and supplements such as treats, vitamins, painkillers, antibiotics, supplements, probiotics, and drug(s) were considered, but these were found to bring in irrelevant mentions such as police dogs and other working dogs, as well as advertisements for nutribiotics for pets. None of the five detailed filters on pill types returned a large volume of posts, making it more challenging to draw meaningful conclusions from the data.

Only 0.2% (*n* = 10) of the SM posts met the coated pills filter. There were nine unique authors, of which two were male, one female, and seven were of unknown gender. Topic modeling found that the most frequently found topic based on post content was “pain” with four mentions.

No posts met the flavored pills filter, while only one post matched the unflavored pills filter.

A total of 7.6% (*n* = 311) of posts met the filter for soft or chewy pills, and those posts had 266 unique authors from the United States. Female authors accounted for 8.3% (*n* = 27) posts, male authors 7.9% (*n* = 23), and unknown gender 83.8% (*n* = 261). Only keywords with over 24 mentions were included in the wordcloud (see Figure 5B), and none of those keywords had a negative sentiment. The keywords “soft” and “medication” had positive sentiment, while “chewable” and “food” had neutral sentiment. This suggests there may be a preference for soft and chewable pill formats.

Looking at the 2.5% of posts (*n* = 101) that mention “chewable” specifically, it is apparent that pet owners often face challenges when administering pills to their dogs, with many turning to chewable medications as a solution due to their ease of use and appeal to pets. Chewable medications are perceived as convenient, as one user noted, “One chewable a month is easy to remember!” and effective, with another mentioning that “Ivermectin is a super safe drug and used all the time for dogs […] They get it as a monthly chewable.” The appeal also lies in the treat-like nature of these chewables, making dogs more likely to consume them willingly, as one owner shared, “Our girl dog has to take a chewable medicine an hour before breakfast, so we have to give the boy dog a treat so he gets something too.” However, chewables are not always universally accepted, with some dogs refusing them despite attempts to hide them in favorite foods, such as “Put it in ham, cheese, and all their other favorites. They ate around it, but the med itself.” Additionally, there are concerns about chewables being too appealing, as another owner expressed worry that their dog could accidentally ingest a large quantity if left within reach, describing how the “tablets [are] yummy on purpose, which is great for ease of administration, but it can obviously cause problems.” The slightly mixed reception highlights the balance between making medications palatable and ensuring safe administration practices among pet owners.

The large dogs’ filter matched 7% (*n* = 292) of posts. These posts were authored by 249 unique authors from the United States. Male authors accounted for 7.6% (*n* = 34) of posts and female authors 5.6% (*n* = 14) of posts. Trending topics were health (*n* = 117 posts), dogs (*n* = 115 posts), and clinical medicine (*n* = 113 posts), while the most frequently mentioned keywords in the dataset were “medication,” “dogs,” and “home.” The majority of keywords in these posts had a positive sentiment, but it was not possible to draw any firm conclusions on discussions around larger dogs and issues around pilling.

### 3.5 Topic modeling outputs

To better understand the wider online narrative surrounding pilling in dogs, we undertook topic modeling and produced topic summaries on original posts surrounding the issues of administering non-flavored, flavored, or chewable pills by pet owners.

Extracted posts were pre-processed as described in the previous section before conducting data-driven topic modeling using NMF on the filtered and pre-processed data. There were 3,630 posts labeled as “relevant” by the classifier for topic modeling. An additional class called “ambivalent” was produced by the classifier (*n* = 160 posts), but these were manually spot-checked, and the majority were found to be irrelevant to the task at hand, such as posts containing advertisements or similar promotions.

Topic modeling generated five topics (themes) along with the top 20 keywords associated with each topic. An LLM approach was adopted to make sense of each of the five ‘word salads’ generated from the topic modeling. These are presented below.

#### 3.5.1 Topic modeling outputs for X, Reddit, blogs, and forums

##### 3.5.1.1 Theme 1: General medication experience and veterinary visits

**Figure 6:**
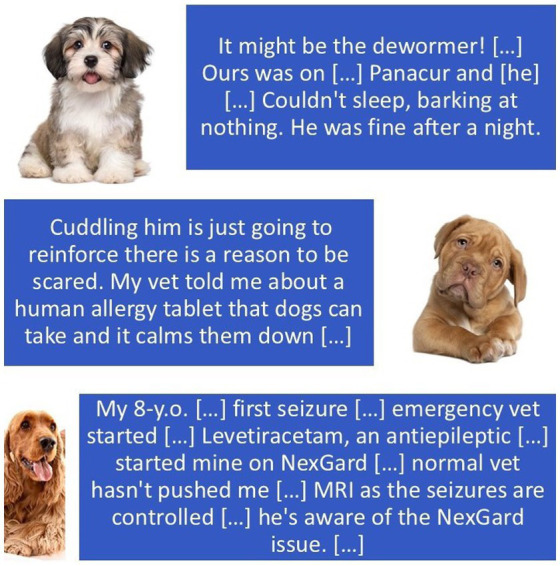
Example social media posts matching topic modeling Theme 1: General medication and veterinary visits.

This theme captures posts concerned with the overall experience of medicating dogs, involving visits to the vet and the general process of administering medication. Common issues discussed include anxiety management, pain relief, and the challenges faced by dog owners when giving medication to their pets. The posts often share personal anecdotes, advice, and experiences about managing their dog’s health, including specific incidents of medicating older dogs or dealing with new medication regimes. Anonymized examples include those shown in Figure 6.

##### 3.5.1.2 Theme 2: Types of pills and general medication practices

**Figure 7:**
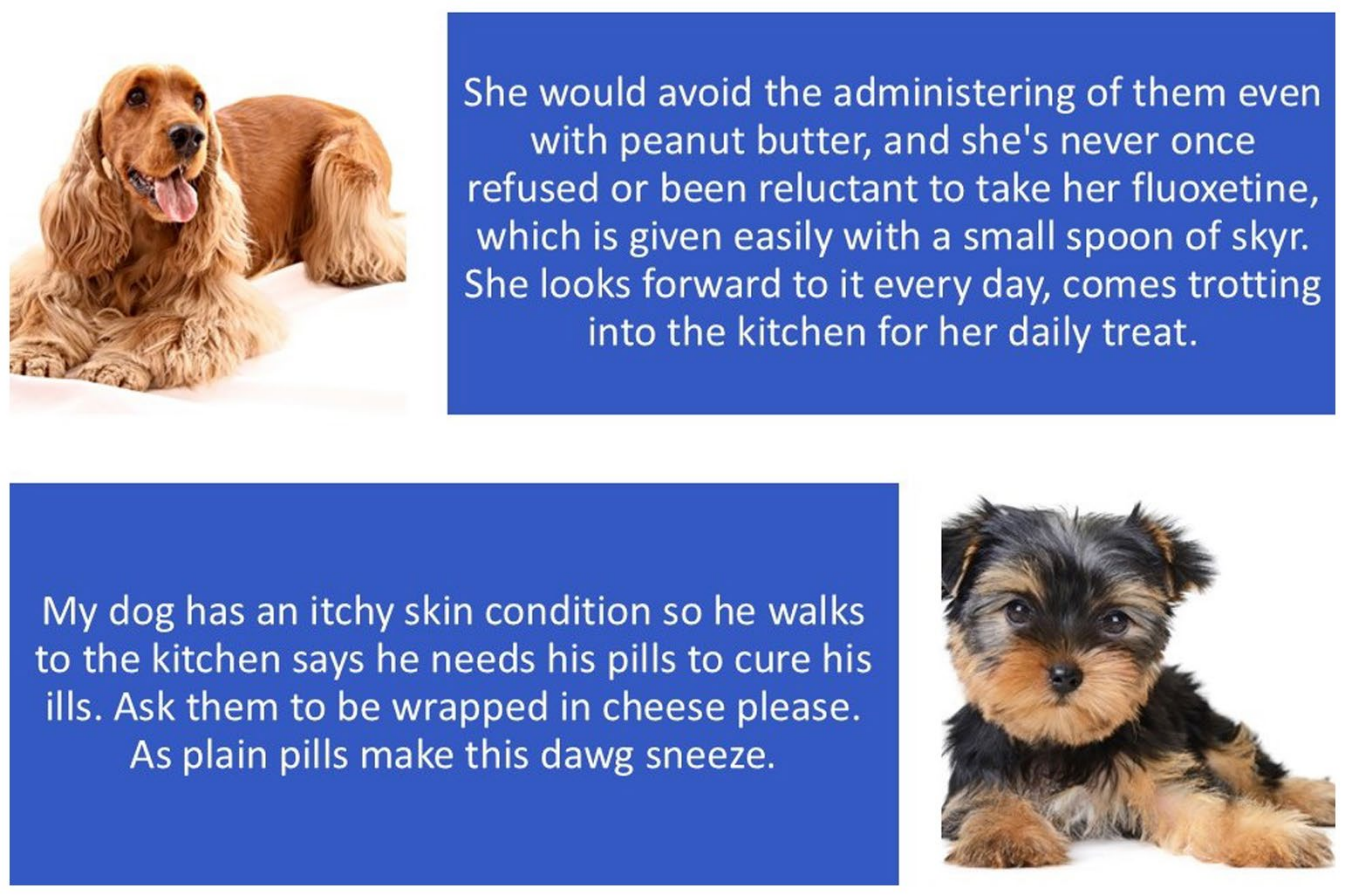
Examples of social media posts matching topic modeling Theme 2: Types of pills and general medication practices.

Posts under this theme (Figure 7) focus on specific types of pills, such as tablets and worm medications, and the general practices involved in giving these pills to dogs. There is mention of different medications, including heartworm prevention and daily medications. Discussions often include the effectiveness of these medications, how well dogs accept them, and tips on making the process easier, such as mixing pills with food to ensure these are consumed.

##### 3.5.1.3 Theme 3: Pill delivery methods

**Figure 8:**
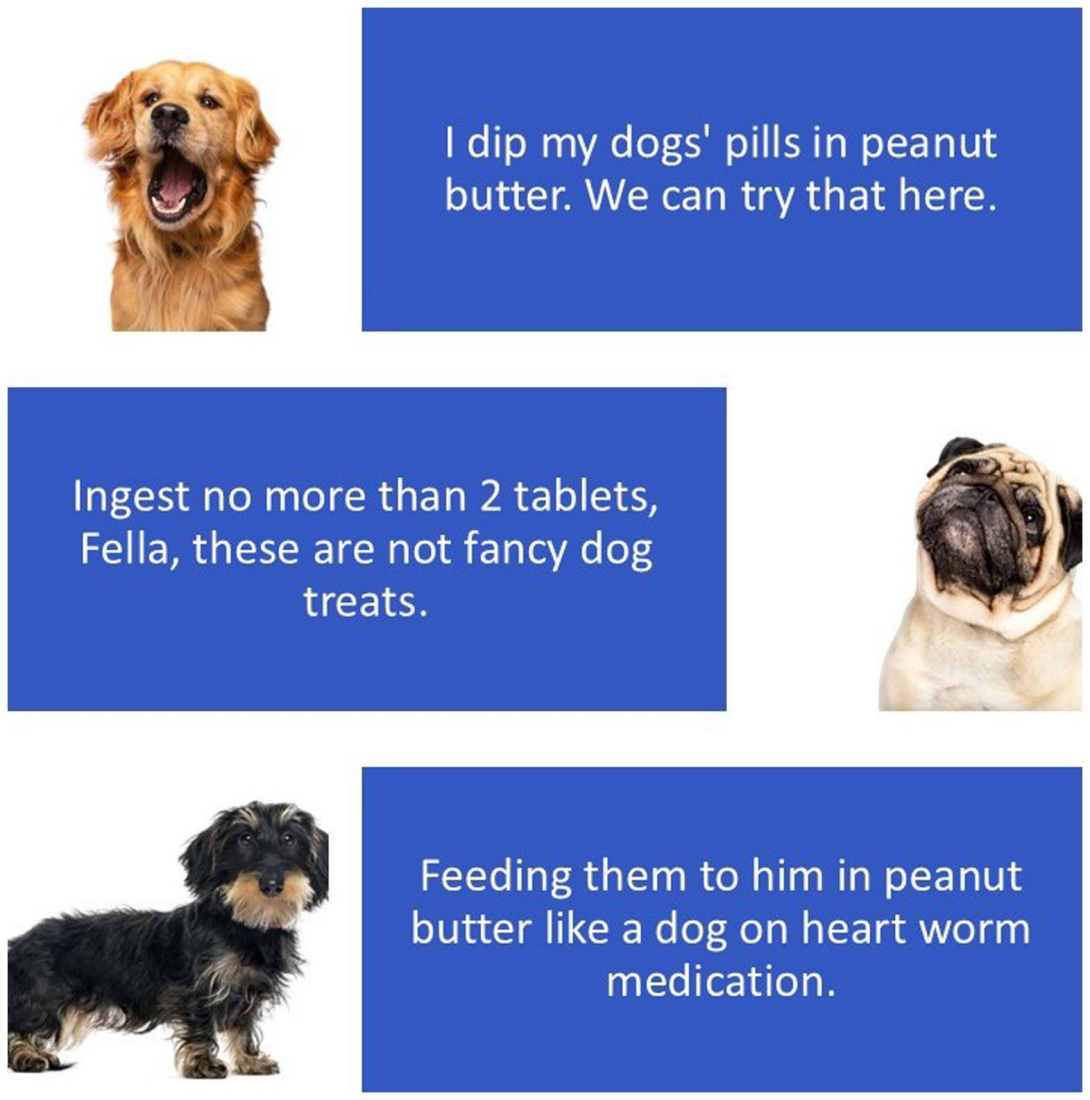
Examples of social media posts matching topic modeling Theme 3: Pill delivery methods and tricks.

This theme is centered around the various methods and tricks used to successfully administer pilling medication. Posts (Figure 8) frequently mention using pill pockets, treats, cheese, chicken, peanut butter, and other creative ways to hide the medication. There is a focus on the practical side of pill delivery, sharing tips and hacks among pet owners to ensure successful dose administration. Table 2 summarizes the successful pilling strategies posted on SM.

**Table 2:** Summary of successful pilling techniques used domestically and posted on social media.

Pilling approach	LLM interpreted strategy	Example post(s)
Peanut butter	Peanut butter is a popular choice for hiding pills due to its strong flavor and sticky texture, which makes it difficult for dogs to spit out the pill.	“…I dip my dogs’ pills in peanut butter. We can try that here.” / “Without even thinking I gave my pup a pill with peanut butter…”
Cheese	American (i.e., processed) cheese is frequently used to wrap pills. The texture helps envelop the pill completely, making it hard for dogs to detect.	“The art of crushing up my dogs pills and melting them into cheese will always amaze me.” / “Do not feel bad, my dogs will not eat edible heartworm medicine and they had to have sharp cheddar not American cheese to take their pills”
Meat or meat products (chicken, lamb, beef etc.)	Wrapping pills in slices of ham, sausage, or other types of meat is another common approach. The strong flavor and scent of the meat can effectively mask the pill.	“…For cats I use a pill pusher. Dogs, wrap it in meat / cheese / peanut butter.” / “gave my dog his pills wrapped in some mortadella and he gobbled it so fast he did not even have time to chew lmao…”
Crushing pills and mixing with food	For dogs who are adept at detecting whole pills, owners often crush the pills and mix them into wet food, cheese, or other strong-smelling foods.	“The art of crushing up my dogs pills and melting them into cheese will always amaze me.” / “My little dog does not want medicines. I crush a pill, mix with water and give him in his mouth with a syringe….”
Distraction and deception	As an example, giving the dog a regular treat first and then quickly following it with the treat containing the pill can sometimes distract the dog enough that they do not notice the pill.	“If you have another dog, give that dog a slice of cheese without medicine, and then give the cheese with meds in it.” / “…Sometimes pills or tablets taste foul when wet, so the poor dogs need something that completely covers the pill without getting wet….”

##### 3.5.1.4 Theme 4: Specific medications and preventative treatments

**Figure 9:**
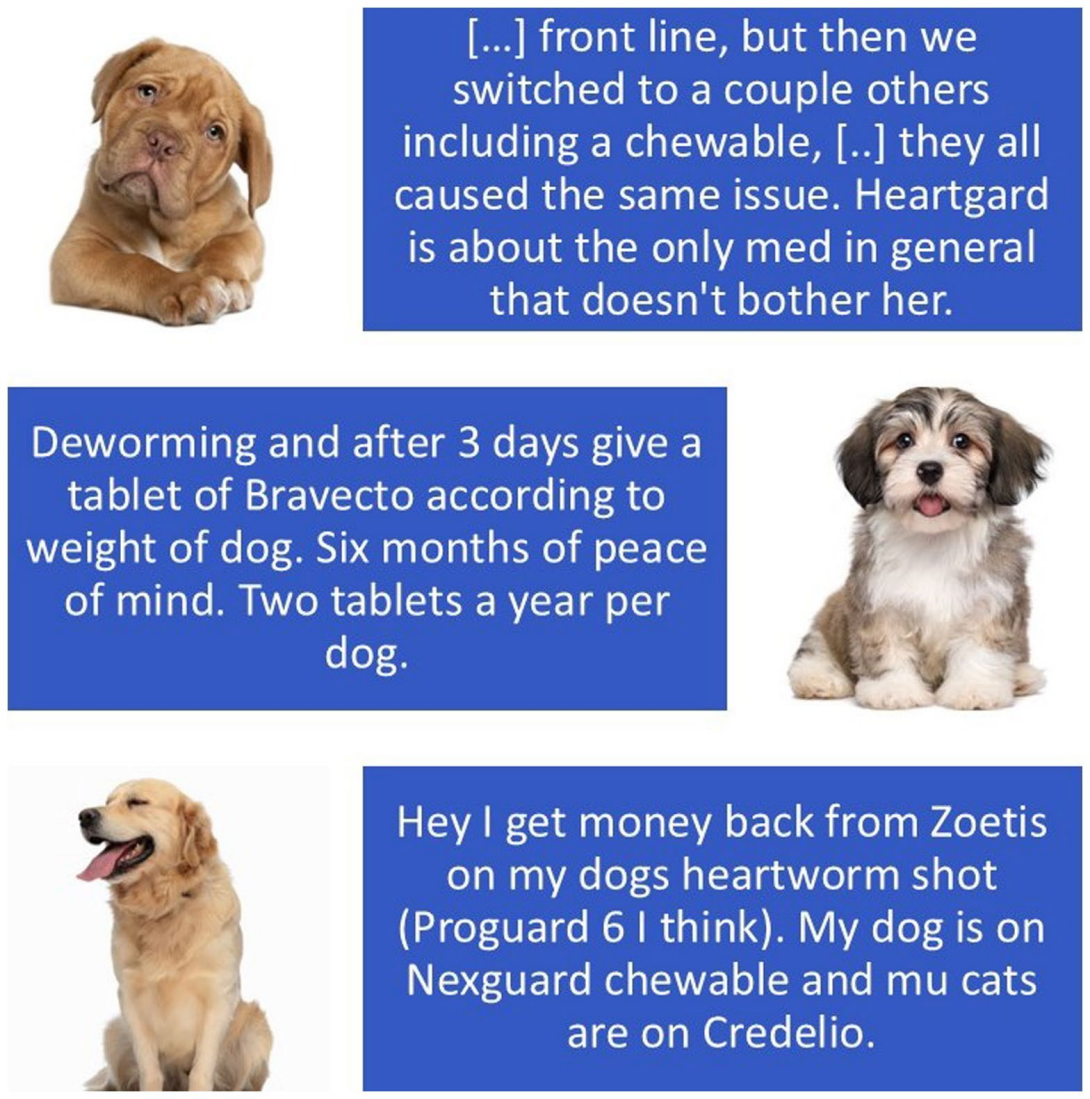
Examples of social media posts matching topic modeling Theme 4: Specific medications and preventative treatments.

Posts in this theme (examples in Figure 9) discuss specific medications and preventative treatments, such as those for ticks, fleas, and heartworm. There is a particular emphasis on chewable tablets and oral medications. The theme covers not only the types of medications but also the routines (e.g., monthly treatments) and safety aspects of using these products.

##### 3.5.1.5 Theme 5: Product reviews and natural remedies

**Figure 10:**
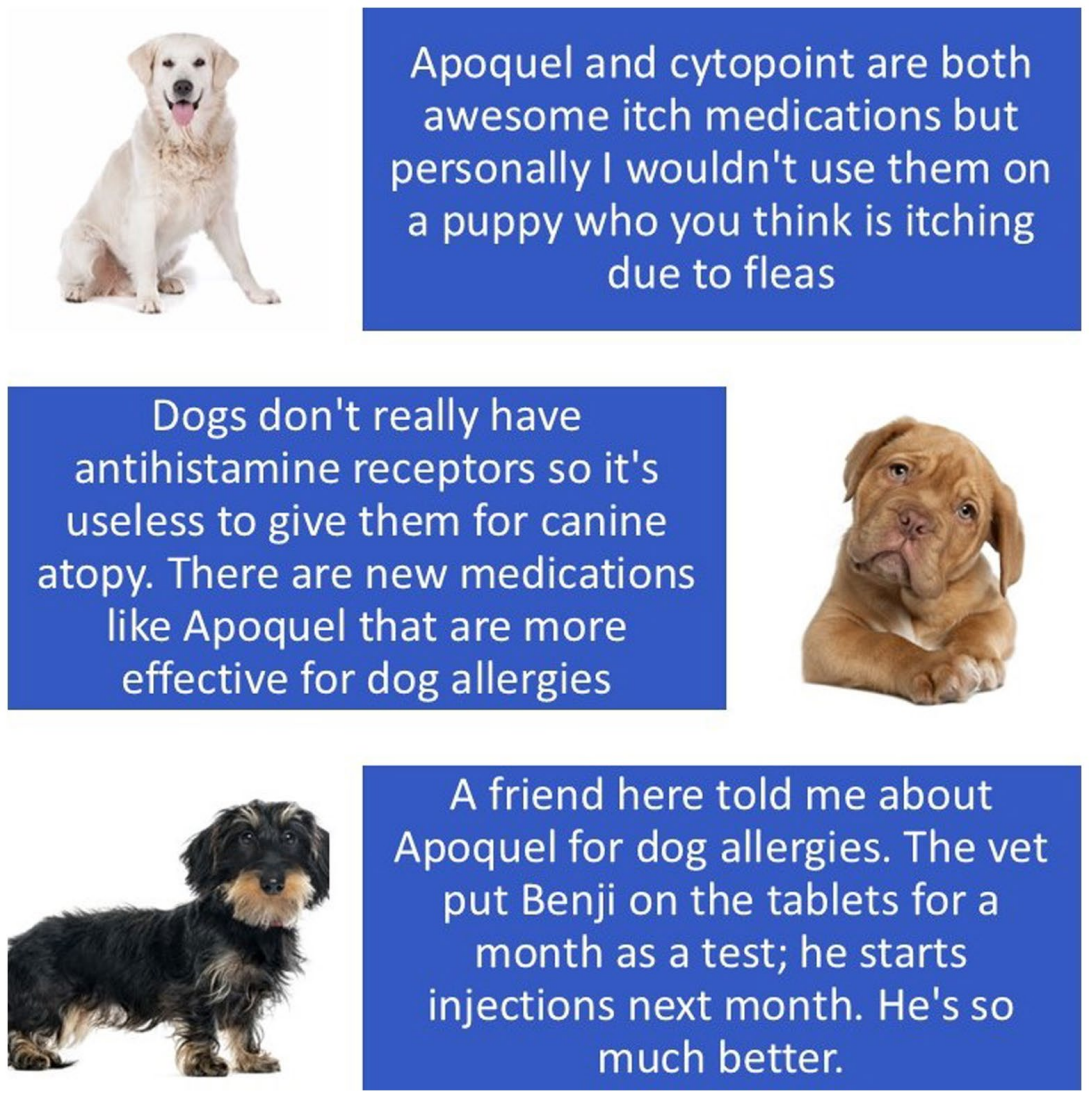
Examples of social media posts matching topic modeling Theme 5: Product reviews and natural remedies.

This theme (Figure 10) includes posts that review specific products, share links to resources, and discuss natural remedies. There is mention of products such as Apoquel (a film-coated or chewable formulation of oclacitinib maleate indicated for the treatment of pruritus) (39). Posts mention various formats of images or links to these products and discussions on natural alternatives such as garlic. The focus is on sharing information about different medication options, including pharmaceutical and natural treatments, and providing opinions on their effectiveness and ease of use.

#### 3.5.2 Topic modeling outputs for Facebook

The same aforementioned topic modeling approach was undertaken using Facebook data. The resulting topic themes are described below:

##### 3.5.2.1 Theme 1: Veterinary and animal care resources

This theme focuses on technical and informational content related to animal care, featuring links to resources (e.g., websites) and discussions about care techniques, adoption information, and veterinary advice.

##### 3.5.2.2 Theme 2: Foster care and adoption stories

This theme indicates a strong focus on the personal and emotional aspects of fostering and adopting dogs. Descriptors such as “home,” “love,” “family,” and “adoption” highlight narratives or testimonials related to the experiences of fostering pets, adopting them into new homes, and the affectionate bonds that develop between dogs and humans.

##### 3.5.2.3 Theme 3: Specific animal care events and logistics

This theme revolves around a specific animal care event where pets can be chipped, vaccinated for rabies and other infectious diseases, and supplied with routine antiparasitics. This is an organized event like a vaccination drive or informational session.

##### 3.5.2.4 Theme 4: Individual pet stories with a health focus

This theme revolves around individual stories of pets with specific health issues or care needs, particularly chronic conditions. Words like “diagnosed,” “cushings,” “I love my medicine wrapped […] cheese,” and “I love […] puzzles” suggest detailed accounts of pets’ health journeys and their daily care routines, which may include unique activities or therapies designed for their comfort and stimulation.

##### 3.5.2.5 Theme 5: Special needs and high-care pets

This theme centers on pets with serious medical needs, as indicated by quotes such as “He was dying from a liver shunt when we rescued him,” “He needs an echocardiogram,” and “Today was a very expensive rescue day with 4 dogs.” These focus on the challenges and processes involved in caring for pets with serious health conditions, including fundraising for medical expenses and the community support required to manage such cases.

#### 3.5.3 Thematic analysis for fear and anxiety

We also undertook thematic analysis using the curated data (*n* = 4,787 posts from Reddit, X, blogs, forums, and Facebook) and AI tools to automate our analysis. The analysis of SM posts reveals the following frequencies of fear and anxiety-related terms:

- **Anxiety**: 592 (12%) mentions
- **Scared**: 128 (3%) mentions
- **Fear**: 96 (2%) mentions
- **Stress**: 116 (2%) mentions
- **Worry**: 83 (2%) mentions
- **Nervous**: 82 (2%) mentions
- **Afraid**: 55 (1%) mentions
- **Panic**: 33 (1%) mentions

The thematic analysis of SM posts reveals the following common themes related to fear and anxiety among dogs and pet owners:

##### 3.5.3.1 Theme 1: Vet visits and medication

Discussions often revolved around visits to the vet, medications, and concerns about how dogs react to these situations. Common words include “vet,” “medication,” and expressions of uncertainty or distress such as “just” and “do not know,” indicating anxiety related to medical care.

##### 3.5.3.2 Theme 2: Home and family environment

This theme highlights the emotional bonds between pets and their families, fostering environments, and the needs of dogs within these settings. Words such as “home,” “love,” “family,” and “foster” point to anxiety related to adjusting to new environments or changes in the household.

##### 3.5.3.3 Theme 3: Feeding and medication administration

This theme focuses on the challenges of administering medication to dogs, particularly pills. Common terms include “pills,” “treats,” “cheese,” and “peanut butter,” suggesting strategies pet owners use to reduce anxiety when giving medication.

##### 3.5.3.4 Theme 4: Tick, flea, and parasite prevention

This theme focuses on flea, tick, and heartworm prevention and associated anxiety about infestations or treatments. Words such as “tick,” “flea,” “heartworm,” and “prevention” indicate worries related to parasites and their impact on pets.

##### 3.5.3.5 Theme 5: Adoption and care

Many posts discuss adoption, care for rescue animals, and the need for assistance. Terms such as “adopted,” “help,” “care,” “rescue,” and “need” suggest anxiety around the adoption process and providing adequate care for pets, especially rescues.

#### 3.5.4 Thematic analysis for financial costs

To further our research, we conducted AI-enhanced thematic analysis using the methodology and datasets (*n* = 4,787 posts) described in Section 3.5.3. When examining these SM posts, we identified the following dominant themes related to the financial costs associated with veterinary care:

##### 3.5.4.1 Theme 1: Veterinary and emergency costs

A number of posts highlight that vet visits and emergency treatments are a major financial burden. Users mention struggles with paying for unexpected surgeries or emergency vet care, difficulty affording routine vaccinations and checkups, and financial strain leading to delays in seeking care or choosing fewer optimal treatments.

##### 3.5.4.2 Theme 2: Medication and long-term treatment

Ongoing medication for chronic conditions such as seizures, allergies, or anxiety was noted as a recurring expense. This includes specialized prescriptions and behavioral medications for anxiety-related issues.

##### 3.5.4.3 Theme 3: Pet insurance and budgeting

Some users discussed pet insurance as a way to mitigate costs but also expressed frustration over the high premiums, exclusions in coverage, or limited reimbursements and expressed uncertainty about whether insurance is worth the cost for aging pets.

##### 3.5.4.4 Theme 4: Pet adoption and initial set-up costs

There were mentions of people considering or delaying adoption due to the adoption fees and costs such as vaccinations.

##### 3.5.4.5 Theme 5: Daily and lifestyle adjustments

Pet owners shared stories of having to cut back on personal expenses to care for their dogs, including skipping travel, delaying personal purchases, and making lifestyle adjustments to account for pet-related expenses.

##### 3.5.4.6 Theme 6: Donation and support appeals

A few posts linked to donation platforms, such as PayPal or GoFundMe, show that some owners (and pet shelters) rely on public support to afford care for their dogs.

While themes 1–6 relate to veterinary medical prescriptions, they lack focus on oral medications. For this reason, the analysis was repeated using a filtered dataset with *n* = 359 posts from Reddit, X, blogs, and forums that mentioned “financial,” “finance,” “cost,” “costs,” or “expensive.” The dominant themes from this analysis were:

###### 3.5.4.6.1 Financial theme 1: Recurring monthly costs

Pet owners struggle with ongoing expenses for basic dog care, sharing specific estimates like “I pay about $30/month for a 22 lb. dog. $10 for heartworm medicine and $8 for flea and tick prevention…,” while comparing premium versus budget options. Essential preventative medications add to the financial burden, along with insurance premiums. These posts highlight the challenge of budgeting for and sustaining these long-term costs.

###### 3.5.4.6.2 Financial theme 2: High medical and treatment costs

The expense of veterinary care represents a major concern, particularly specialist visits, advanced diagnostics (blood work, imaging), and daily medications for chronic health or behavioral issues. Many express shock at how quickly vet bills accumulate, even for basic care, creating significant financial stress from unexpected or ongoing medical needs.

###### 3.5.4.6.3 Financial theme 3: Cost of supplements and health products

Owners frequently mention supplements and alternative health aids for senior dogs or those with special needs. While these products enhance the quality of life (QoL), they are framed as financially burdensome. Posts demonstrate how owners are willing to spend more on their pet’s wellbeing despite the financial strain this causes.

###### 3.5.4.6.4 Financial theme 4: Perception of expense as a barrier to ownership

Comments questioning whether certain products or treatments are “too expensive” or necessary reveal deeper concerns about affordability. Many owners’ resort to seeking cheaper alternatives or making compromises due to financial constraints, suggesting that these barriers may ultimately deter optimal pet care or even pet ownership altogether.

#### 3.5.5 Thematic analysis for perceived benefits of treatment

Additional thematic analysis was conducted to investigate whether the pet owner’s perceived benefit of treatment was a contributing factor in veterinary medicines compliance. As before, we used AI tools to analyze *n* = 4,787 posts from Reddit, X, blogs, forums, and Facebook. The dominant themes found in the data that were most relevant to our study (see section 1.1. of the Supplementary materials for all themes and theme descriptions) were:

- *Anxiety and behavioral treatments*
- *Home life and emotional support*
- *Medical issues and specialized diets*

The extracted themes using the full curated dataset were related to veterinary medicines but with a broader scope than oral medications alone. Therefore, further analysis was conducted on a filtered data sample containing posts mentioning keywords relating to the positive benefits and the negative side of treatment. There were 1,417 posts (40.5% of the full dataset) in the positive benefits sample and 807 (23%) in the negative sample. The following themes were extracted for the positive benefits of treatments as perceived by pet owners:

##### 3.5.5.1 Effective parasite control (fleas, ticks, and heartworm)

Pet owners frequently praised medications for their efficacy in controlling external and internal parasites such as fleas, ticks, and heartworm. Products such as monthly chewable tablets are highlighted for maintaining the health of both dogs and cats, offering convenience and reliable protection.

##### 3.5.5.2 Emotional bonding

Medications are viewed as lifesaving and enabling deeper human–animal bonds. Owners shared emotionally rich stories of adopting pets with health conditions and successfully managing those conditions, reinforcing their connection and mutual trust.

##### 3.5.5.3 Chronic condition management and improved quality of life

Long-term treatments for conditions such as arthritis, Cushing’s disease, and allergies are seen as dramatically improving pets’ comfort and behavior. Many users report that proper medication restored their pets’ personalities, reduced pain, and reversed weight loss.

##### 3.5.5.4 Daily management and owner resilience

Owners describe the mental and physical effort of daily care for medicated pets: administering doses, monitoring symptoms, and adjusting routines. Despite challenges, they express deep satisfaction in seeing their pets recover or maintain a good QoL.

The following dominant themes were extracted for the negative aspects of treatments as perceived by pet owners:

##### 3.5.5.5 Ineffectiveness and trial-and-error of treatments

Many users report frustration with the unpredictability and inconsistency of pet treatments. Owners often mention trying multiple medications or methods without reliable results.

##### 3.5.5.6 Anxiety, pain, and emotional distress in pets

A recurring theme involves treatments leading to or failing to alleviate anxiety, pain, or distress, especially in dogs. Owners describe how treatments do not address root causes and sometimes even exacerbate the discomfort.

##### 3.5.5.7 Concerns over over-medication and side effects

Several posts mention the concerns about the overuse of medications, harsh side effects, and a preference for more natural or holistic approaches.

##### 3.5.5.8 Financial strain and accessibility issues

Treatment costs are a major burden. Pet owners frequently reference the high expense of vet visits, medications, and emergency care, making them question the value or accessibility of some treatments.

##### 3.5.5.9 Mistrust toward vets and pharmaceutical advice

There’s a strong undercurrent of mistrust toward veterinary advice, particularly when treatment seems profit-driven or when multiple vet opinions contradict each other.

##### 3.5.5.10 Emotional toll on pet owners

Owners often describe the emotional burnout and sadness involved in managing chronic illness in their pets, especially when treatments are not effective. This is more prominent among those with long-term caregiving roles.

## 4 Discussion

### 4.1 Effectiveness of social media listening and large language models in capturing real-world pet owner experiences

This study demonstrated the utility of using SML and AI-led analysis using LLMs in capturing and analyzing real-world descriptions of the experiences of pet owners when administering oral medications to their dogs. By leveraging a commercial social listening platform for data scraping with targeted search terms, the study successfully gathered a substantial dataset from diverse SM sources, including X, Reddit, Facebook, blogs, and forums. These searches were intended to be broad enough to bring in sufficient posts for valid statistical analysis while being sufficiently narrow to minimize noise from irrelevant posts.

The subsequent use of AI tools, including LLMs for data cleaning and filtering, ensured that the dataset was relevant and manageable, enabling a focused analysis of pet owner challenges in administering oral medications to dogs.

The application of LLMs for zero-shot relevancy filtering proved to be a compelling advancement over manual classification methods. This approach not only increased efficiency but also enhanced the accuracy of the analysis, as evidenced by the successful identification of relevant posts despite the initial presence of noise in the dataset, such as duplicate content and unrelated posts. This study’s methodology highlights the potential of LLMs in large-scale SM research, particularly in fields where manual data handling would be impractical due to the volume of data.

We found that the data scraped across different platforms had varying levels of utility. This may be reflective of platform-specific behaviors/formats. Posts from Facebook were of limited value in providing insights into veterinary issues. This largely consisted of positive dog-related stories rather than individuals seeking help or solutions provided. Such information may exist on this platform, but if so, it is likely hidden in private groups that are inaccessible to SML platforms. X appears to provide important content and, by the nature of the platform, maybe more closely associated with the posting of problems and issues. Similar blogs and forums, such as Reddit, often contain long stories but with problems and solutions posted alongside, which, along with X, appear to be a useful source of information on pet owners’ real-world experiences.

### 4.2 Comparison of oral medication types

The findings from this study reveal distinct challenges associated with different types of oral medications for dogs. A prominent challenge is that despite a number of different pilling types/offerings, flavored treatments are often preferred (1, 25), but this alone may not be a panacea for medication adherence issues. The topic modeling outputs for X, Reddit, blogs, and forums revealed several pill delivery techniques (Theme 3: Pill delivery methods), including the use of pill pockets and/or treats to hide pills, chicken or strong-smelling foods such as cheese and peanut butter to entice reluctant dogs to eat crushed pills. However, it should be noted that oral products are carefully formulated to ensure reliable and predictable drug dissolution, absorption, and performance, and the impact of an owner crushing, powdering, mixing, or hiding the medication with foods/liquids, etc. (as in Table 2. Summary of successful pilling techniques used domestically and posted on social media.) may alter drug performance. The impact of this is largely unknown; therefore, incorporating easy-to-give characteristics into the product at the development phase removes such uncertainty of outcome. The findings underscore the need for continued innovation in medication formulation, potentially incorporating advanced flavor enhancers or novel delivery systems to improve compliance.

### 4.3 Geographical and demographic variations

The geographic analysis of SM posts discussing the administration of oral medications to dogs reveals an international presence across a number of countries on these topics, unsurprisingly dominated by the United States contributing the majority (*n* = 445, 70.3%), followed by the United Kingdom (*n* = 144, 17.8%), Australia (*n* = 81, 7%), and Canada (*n* = 44, 5%). This distribution reflects the language of the search terms, which were in English, and possibly the higher prevalence of dog ownership in these regions. Posts are predominantly located in urban areas, likely due to the larger human and pet populations in cities, which could skew the geographic distribution toward these areas. There was no particularly noticeable difference in the ratios of men and women posting on these issues.

Sentiment analysis of these posts indicates a generally positive outlook among pet owners when discussing medication administration to dogs. Positive sentiments were particularly associated with keywords such as “medication,” “home,” and “help,” suggesting a supportive and solution-oriented discourse. However, negative sentiments emerged in discussions involving difficulties with pill administration, highlighted by words such as “pills,” “bad,” “eat,” and “trying.” Interestingly, mentions of “pain” and “tablets” remained neutral, reflecting a more factual and less emotionally charged tone compared with other topics on SM. Overall, while the sentiment leans positive, there is a meaningful portion of posts reflecting the challenges and frustrations of medicating dogs, especially regarding pill forms and large dog breeds. A marginal predominance of male dog owners posting about large dog breeds was observed, though the limited sample size precludes definitive conclusions. Previous research studies have shown that men frequently select larger, more athletic dog breeds, as these choices often reflect the owner’s self-image and identity (38).

### 4.4 Non-compliance with preventative, short, and long-term medications

Adenot et al. and Lavan et al. both identified pet owner-related factors as major causes of treatment non-compliance, including administering doses at incorrect times, outside optimal efficacy intervals, or creating gaps in medication purchase (1, 2). Posts in Theme 4: Specific medications and preventative treatments (section 3.5.1) captured the online discourse on the topic of chewable and other oral medications, the safety of these products, and dosing routines, suggesting that pet owners are seeking support from other pet owners. Pet owners may demonstrate higher compliance when treating discernible and/or chronic conditions that visibly impact the pet’s QoL, when treatments are of short duration (such as antimicrobial courses), or when they fully comprehend the prescribed treatment protocol (3, 4, 15). Communication and education are, therefore, fundamental to improving compliance across all veterinary care areas.

A study by Odom et al. reported that 95% of pet owners surveyed claimed to understand the prescribed treatments either “very well” or “extremely well,” suggesting effective vet-client communication. However, Adams et al. discovered that while initial antimicrobial compliance rates were high (as measured by container openings), they declined sharply when daily dosing exceeded twice daily, a finding later corroborated by Odom et al. (3, 4). Both studies recommended less frequent dosing regimens to improve compliance, with Odom et al. demonstrating the effectiveness of combining this approach with a long-acting ectoparasiticide (3). The latter study also concluded that adequate veterinary medication instructions are crucial to enhancing pet owner compliance (3). A review by Manasa et al. found long-acting formulations to be effective in treating numerous animal health conditions across species, including chronic conditions, pain management, novel antiparasitics, and the reduction of anxiety and stress. The authors also postulated that long-acting medications have the potential to transform veterinary care by improving treatment compliance and reducing the challenges pet owners face when administering medications to their pets (40).

Preventative treatments, particularly antiparasitics, typically exhibit lower compliance rates because pet owners often fail to recognize their necessity (12, 30, 31). Although our data did not explicitly reveal pet owner reluctance toward antiparasitics, we identified “a strong undercurrent of mistrust toward veterinary advice, particularly when treatment seems profit-driven” that may be related. Conversely, some pet owners discussed “concerns about flea, tick and heartworm prevention and associated anxiety about infestations or treatments” on Facebook (in Section 3.5.4, Theme 4: Tick, flea, and parasite prevention), using “words [that] indicate worries related to parasites and their impact on pets.” Veterinarians can improve pet owner compliance with flea and tick prevention through several methods: offering financial incentives such as vouchers and discounts, implementing smartphone reminder systems, enhancing parasite education, and recommending extended-duration parasite control products (2). According to a recent study, approximately 50% of pet owners stated they received no demonstration on medication administration, while one-third encountered difficulties, primarily due to resistant pets (3).

Prolonged medication regimens can significantly diminish treatment efficacy over time. Research has demonstrated that extended pharmaceutical use often leads to the development of drug resistance (21, 40). While antimicrobial resistance is widely recognized, resistance to antiparasitic treatments poses serious concerns. For instance, fipronil-resistant flea populations have emerged, drastically reducing the efficacy of these once-reliable treatments (22). This reality is reflected in pet owner experiences: “We adopted a stray kitten and have treated her twice with Kitten Frontline. On the skin not the fur. I have two other cats and a dog. Never have I had a pet resistant to treatment! We have bathed her twice, applied medication twice and the fleas persist.” This pattern extends to veterinary medicine, where extended or improperly managed antibiotic courses in dogs treating chronic conditions such as recurrent skin infections contribute to antimicrobial resistance (21). Similarly, long-term monthly fipronil administration has been selected for resistant flea strains, and resistant heartworm-causing Dirofilaria immitis populations have developed, undermining previously effective therapeutic approaches (23).

For pets with chronic conditions, the positive response to treatment, as evidenced by the pet owner’s perceived benefits analyzed in Section 4.6, suggests that demonstrable improvements to a pet’s QoL are more likely to encourage treatment adherence. Odom et al. found that senior dogs had significantly higher medication compliance rates. This could be due to older pets having more medical conditions requiring treatment and older pets being more used to receiving treatment. Owners will likely have gained more experience administering medications and may have higher awareness and understanding of the importance of medication compliance for their pet (3), although older pets visit their veterinarian for preventatives less frequently than their younger counterparts (13). Chronic conditions lead to more frequent veterinary interactions, thereby creating more opportunities for vet–client communication and enhancing compliance (13). Social pressures may also apply with pet owners being compelled to care properly for visibly unwell older pets. Conditions such as osteoarthritis may require major changes in daily routine and introduce strong motivation for compliance (3).

### 4.5 Financial factors affecting compliance

Finance is a barrier for many pet owners that “may ultimately deter optimal pet care or even pet ownership altogether” (Financial theme 4: Perception of expense as a barrier to ownership). Purchase compliance is thought to be low (11) as fewer doses are sold compared to veterinarian recommendations (5, 6, 18), “many [pet] owners’ resort to seeking cheaper alternatives” (financial theme 4), and some pet owners are unable to afford treatment. Financial theme 1: Recurring monthly costs (in Section 3.5.4) are concerned with pet owners’ financial challenges paying for essential dog care, including antiparasitics. Although these are an essential part of responsible pet ownership (12), they “add to the financial burden.” Fortunately, “posts demonstrate how owners are willing to spend more on their pet’s wellbeing; despite the financial strain” (Financial theme 3: Costs of supplements and health products).

Emergency care (Section 3.5.4, Theme 1: Veterinary and emergency costs) is another “financial strain leading to delays in seeking care or having to choose less optimal treatments.” The recurring expenses incurred when caring for a pet with a chronic condition (Theme 2: Medication and long-term treatment in section 3.5.4) also cause financial pain. Pet owners “express shock at how quickly vet bills accumulate, even for basic care, creating significant financial stress from unexpected or ongoing medical needs” (Financial theme 2: High medical and treatment costs). “Pet insurance [can] mitigate costs,” but pet owners “expressed frustration over the high premiums” and question “whether insurance is worth the cost for aging pets (Theme 3: Pet insurance and budgeting in Section 3.5.4).

A sick pet leads to “making lifestyle adjustments” including cutting back on other expenses to pay veterinary fees (Theme 5: Daily and lifestyle adjustments, section 3.5.4). Crowd funding is also mentioned (Theme 6: Donation and support appeals, section 3.5.4) with “posts linked to donation platforms like PayPal or GoFundMe.” Implementing strategies like monthly payment options and personalized yearly care plans for pets can help overcome financial obstacles to veterinary treatment (13, 14), particularly with routine preventative treatments such as antiparasitics and annual vaccinations.

### 4.6 Pet owner perceived benefits of treatment

Pet owners are more likely to adhere to treatment if that treatment visibly improves the pet’s QoL (14). Improvements to a pet’s overall QoL are just one of the pet owner’s perceived benefits of treatment as found in our “Perceived benefits” dataset. Demonstrable improvements, such as “medications are viewed as lifesaving” (Emotional bonding theme), indicated that a positive response to treatment is more likely to encourage treatment adherence for pets with chronic conditions. This is reflected in the “Daily management and owner resilience” theme where pet owners said that “Despite challenges, they express deep satisfaction in seeing their pets recover or maintain a good [QoL].”

“Home life and emotional support” and “Medical issues and specialized diets” were dominant themes in the full dataset (Reddit, X, blogs, forums, and Facebook, as described in Section 3.5.5). These themes are likely to be related to the management of chronic conditions rather than short-term or preventative treatments. Ongoing treatments for conditions such as arthritis, Cushing’s disease, and allergies are viewed as “dramatically improving pets’ comfort” with “Many users report[ing] that proper medication restored their pets’ personalities, reduced pain and reversed weight loss.” (Chronic condition management and improved quality of life theme, Section 3.5.5). Caring for a pet requiring daily medications is a “mental and physical effort […] administering doses, monitoring symptoms, and adjusting routines,” meaning that pet owners must develop resilience (Daily management and owner resilience, Section 3.5.5).

Preventative measures such as antiparasitics are also seen as beneficial, with “Pet owners frequently praised medications for their efficacy in controlling external and internal parasites” and “monthly chewable tablets are highlighted for maintaining the health of both dogs and cats, offering convenience and reliable protection” [3.5.5 Effective parasite control (fleas, ticks, and heartworm)].

### 4.7 Negative aspects of treatment as perceived by pet owners

Negative perceptions of treatment included “frustration with the unpredictability and inconsistency of pet treatments” where veterinarians try “multiple medications or methods without reliable results.” (Ineffectiveness and trial-and-error of treatments theme, Section 3.5.5). “Mistrust toward vets and pharmaceutical advice” was of particular note, especially “when multiple vet opinions contradict each other” (Mistrust toward vets and pharmaceutical advice theme, Section 3.5.5).

Our data illustrated pet owner concerns about “overuse of medications, harsh side effects…” (Concerns over over-medication and side effects theme, section 3.5.5). This is consistent with Odom et al. (3), who found that some pet owners stopped treatment after their pet showed signs of potential side effects such as diarrhea (3).

The “Emotional toll on pet owners” theme (section 3.5.5) was poignant as pet owners caring for chronically ill pets “often describe the emotional burnout and sadness involved in managing chronic illness in their pets, especially when treatments are not effective.”

### 4.8 Implications for veterinary professionals and pharmaceutical companies

Studies by Lavan et al. and Mwacalimba et al. reveal a considerable gap between recommended parasiticide treatment and actual oral prevention coverage (7–9, 19). To improve adherence, veterinary professionals should emphasize year-round protection to pet owners and recommend longer-acting medications, which have improved compliance (18, 19). However, recommendations by country vary, and a risk-based approach may be preferred over continuous dosing where the parasite is not endemic (20). Additionally, addressing the tendency of owners to purchase only one dose annually can enhance treatment outcomes and overall canine health.

One alternative to pilling is via some form of injection, which may be potentially long-lasting, thereby overcoming the issues of regular pill dosing needed and associated issues with prescription compliance (41). However, not all dogs are a good fit for injectable treatments such as heartworm preventatives (8–10). Although we did not explicitly use words associated with injections as part of our search strategy, we naively proposed that this may arise as part of the discussions around pilling and pilling alternatives. However, in the dataset used in this analysis, there were few mentions of injections or injectables in the posts (e.g., ProHeart 6, a 6-monthly vet-administered injectable to prevent Heartworm disease in dogs, manufactured by Zoetis Inc., was mentioned, Figure 9). This suggests either little understanding of alternative drug administration methods or no particular desire to seek alternatives to domestically administrated pilling.

### 4.9 Limitations

As with any study that uses a sample population to understand the wider population, there are potential limitations associated with sampling methodology. Reliance on SM data collection introduces potential biases as the personality types that choose to post on SM may not represent the broader pet owner population. Nonetheless, given the popularity and propensity of SM in modern daily lives and the non-controversial nature of the topics discussed here, such risks are thought to be minimal. While our comprehensive multi-platform approach captured a rich diversity of experiences, future research could enhance this methodology by developing sophisticated techniques to cluster posts from the same author that would allow for even more precise analysis by accounting for multiple submissions potentially originating from the same individuals across different platforms, potentially reducing overrepresentation of certain experiences or incidents. The development of search expressions focused on the topic of dog pilling may have limited the dataset’s comprehensiveness. To mitigate this, we specifically tried to optimize sensitivity over specificity in the initial data scrapes and then filtered the resulting data off-platform for relevancy, etc. Additionally, constraints on Facebook’s search feature, specifically the search syntax and the limit to 20 keyword combinations per search, plus the need to filter out unwanted content, may potentially have led to the exclusion of some relevant posts. The Facebook restriction meant that to be practicable, and multiple searches had to be run that focused on the most popular large dog breeds and mentions of dogs in general as opposed to searching for all large dog breeds, mentions of large dogs, and breeds that come in large (standard) size. This will not have unduly affected the results, as irrelevant posts should have been filtered out by the zero-shot filter.

### 4.10 Future work

Future research should consider broadening the demographic scope to include non-English language posts, providing an expanded global perspective on pet owner challenges in administering medications. Expanding the scope could also aid in creating a more comprehensive dataset. Conducting longitudinal studies to track changes in attitudes and experiences over time will offer deeper insights into evolving trends in the landscape of veterinary care and pet medication administration. Incorporating image analysis alongside text analysis, which is now feasible with the latest (in 2024) generation of LLMs, could enhance the understanding of SM content while comparing findings with traditional survey methods could validate the results.

Exploring real-time SM monitoring could aid in the early detection of medication-related issues or trends, and applying similar methodologies to study medication administration in other companion animals, such as cats, will expand the research’s applicability. Developing targeted interventions based on these findings can improve medication adherence, ultimately leading to better health outcomes for pets. Additionally, further refining relevancy filters and addressing issues such as unwanted content in SML studies will enhance data quality and reproducibility. Developing and testing targeted interventions based on the findings to improve medication adherence would be a logical next step in this research.

Unforeseen content matches that are irrelevant to the present study are almost inevitable in many SML studies. Future work to aid researchers in removing this kind of content would be of great utility.

## 5 Conclusion

This study provides key insights into the challenges pet owners face in administering oral medications to their dogs. We demonstrated that of those encountering problems, many report issues of fear and anxiety about the pilling process. We have found that chewables can be more popular and easier-to-use in some cases, but there is a fear of over-dosing if the pills are seen as too appetizing. This may, in part, be mitigated by proper storage of these medications including the use of a suitable, safe delivery point for home delivery of medications.

**Figure 11:**
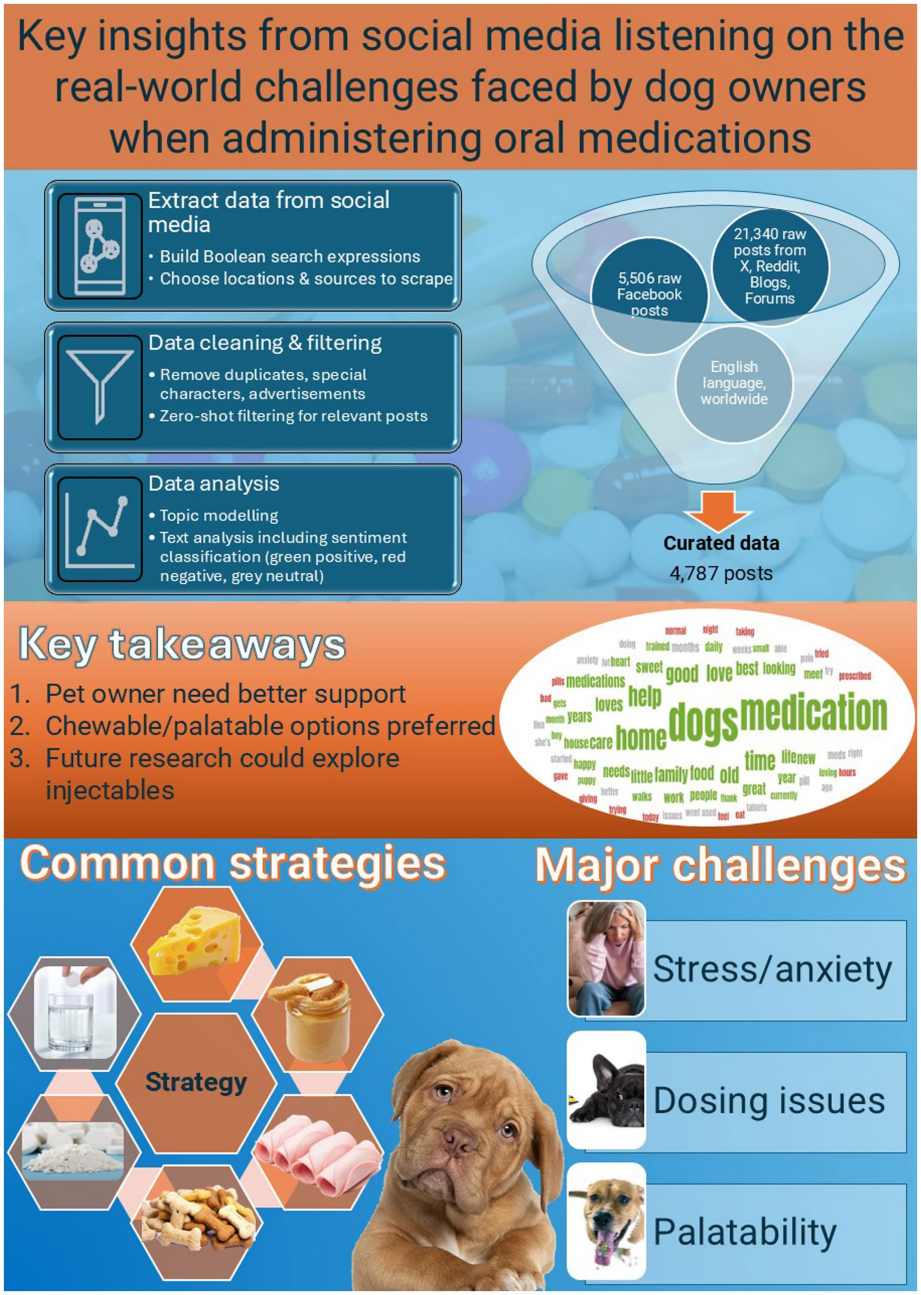
Infographic summarizing the overall methodology, social media data collection, sentiment analysis, major challenges for pet owners when administering oral medications, and common strategies for success, as well as key takeaways from the study.

Our SML data has also been used to compile a list of the most successful strategies used to successfully administer oral medication. This may be useful for vets and owners alike. Successful pilling approaches adopted by pet owners included hiding pills in peanut butter, cheese, meat, or meat products such as chicken, lamb, or beef; crushing pills and mixing them with food; or distraction and deception techniques like feeding the dog a regular treat before feeding a treat containing a pill (see Figure 11). Veterinarians may wish to default to chewable or palatable formulations when they are available or consider alternatives to oral medications when longer-lasting injectables are indicated and available to reduce pet owner anxiety and stress.

The novel methodology has proved valuable in uncovering real-world data, complementing traditional research methods such as surveys and focus groups. Such work might also inform and shape questionnaires on specific veterinary topics.

## Data Availability

The data supporting the conclusions of this article will be made available by the authors, without undue reservation.

## Footnotes

^1^A large language model (LLM) is a machine learning model that has been pre-trained on vast volumes of data. LLMs can recognize and generate text and perform other natural language processing tasks such as classification (28).
