# Supplementary material for: Using social media listening to identify the real-world challenges faced by dog owners globally when administering oral medications
Source: Front Vet Sci. 2025 Jun 30;12:1502236. doi: 10.3389/fvets.2025.1502236 (PMC12258389; doi:10.3389/fvets.2025.1502236)
Supplement: Supplementary file 1 [file Table_1.docx]

Supplementary Material

Using social media listening to identify the real-world challenges faced by dog owners globally when administering oral medications

Georgina Tarrant^1*^, Taranpreet Rai^1,2^, Andrea Wright^3^, Travis Street^1^, Kevin Wells^1,2^

^1^Surrey DataHub, vHive, School of Veterinary Medicine, University of Surrey, Guildford, United Kingdom

^2^Centre for Vision, Speech and Signal Processing, University of Surrey, Guildford, United Kingdom

^3^Outcomes Research, Zoetis Inc., Parsippany, NJ, United States

*** Correspondence:**Georgina Tarrant
georgina.tarrant@surrey.ac.uk

# Supplementary Data

## Themes relating to pet owner perceived benefits and negative aspects of veterinary treatment

Additional thematic analysis was conducted to investigate whether the pet owner’s perceived benefit of treatment was a contributing factor in veterinary medicines compliance. As for the financial costs analysis, we used AI tools to analyse n=4,787 posts from Reddit, X, blogs, forums and Facebook. The dominant themes that were most relevant to our study were extracted from the data.

*1.1.1 Theme 1: Rescue and adoption*

Posts frequently describe treatments in the context of newly adopted pets, particularly cats. Users share stories about establishing care routines and addressing any early-stage medical or behavioral needs that arise during the transition period.

*1.1.2 Theme 2: Anxiety and behavioral treatments*

A significant number of pet owners discuss interventions for managing anxiety and behavioral issues, especially for dogs. These conversations often detail medication options alongside training strategies, highlighting the combined approach many take to address their pets' emotional well-being.

*1.1.3 Theme 3: Charity and awareness campaigns*

Treatment discussions sometimes appear within fundraising contexts, where users promote specific products or interventions as part of broader awareness initiatives or rescue support campaigns.

*1.1.4 Theme 4: Home life and emotional support*

Many posts frame treatments within the context of the pet-owner emotional bond, emphasizing how interventions improve quality of life and strengthen relationships at home.

*1.1.5 Theme 5: Medical issues and specialized diets*

Specific health conditions, particularly liver issues and their management, feature prominently in discussions. Posts detail dietary interventions and medical treatments prescribed for conditions like liver shunts, with specialized feeding requirements.

The themes extracted from the full curated dataset were considered too broad therefore a further analysis was conducted on a more targeted data sample of posts mentioning keywords relating to the positive benefits of treatment and the negative aspects of treatment.

## Themes relating to pet owner perceived benefits of veterinary treatment

Further thematic analysis was undertaken using a filtered sample of posts mentioning keywords relating to the positive benefits of veterinary treatment as perceived by pet owners. There were 1,417 posts (40.5% of the full dataset used in section 1.1) in the positive benefits sample. The following themes were extracted relating to the positive benefits of treatments as perceived by pet owners:

*1.2.1 Theme 1: Effective parasite control (fleas, ticks, heartworm)*

Pet owners frequently praised medications for their efficacy in controlling external and internal parasites such as fleas, ticks, and heartworms. Products like Bravecto and monthly chewable tablets are highlighted for maintaining the health of both dogs and cats, offering convenience and reliable protection.

*1.2.2 Theme 2: Emotional bonding and rescue outcomes*

Medications are viewed as lifesaving and enabling deeper human-animal bonds, especially in the context of foster care and rescue. Owners shared emotionally rich stories of adopting pets with health conditions and successfully managing those conditions, reinforcing their connection and mutual trust.

*1.2.3 Theme 3: Chronic condition management and improved quality of life*

Long-term treatments for conditions like arthritis, Cushing’s disease, and allergies are seen as dramatically improving pets’ comfort and behavior. Many users report that proper medication restored their pets’ personalities, reduced pain, and reversed weight loss.

*1.2.4 Theme 4: Community-based medical support and fundraising*

There is a strong theme around community-driven medical aid whereby volunteers and organizations actively fundraise and provide treatment for rescued or injured animals. Treatments like Carprofen for pain relief are emphasized, along with public appeals for ongoing support.

*1.2.5 Theme 5: Rescue organization campaigns for treatment access*

European (notably Hungarian) rescue groups promote the benefits of medical treatments as part of broader campaigns for adoption and financial support. They share stories of how treatments have stabilized critically ill or neglected animals, making them adoptable and healthy.

*1.2.6 Theme 6: Daily management and owner resilience*

Owners describe the mental and physical effort of daily care for medicated pets: administering doses, monitoring symptoms, and adjusting routines. Despite challenges, they express deep satisfaction in seeing their pets recover or maintain a good quality of life.

## Themes relating to pet owner perceived benefits of veterinary treatment

To complete the thematic analysis described in section 1.2, further thematic analysis was undertaken using a filtered sample of posts mentioning keywords relating to the negative aspects of veterinary treatment as perceived by pet owners. There were 807 posts (23% of the full dataset used in section 1.1) in the sample. The following themes were extracted relating to the negative pet-owner perceived view of veterinary treatments:

*1.3.1 Theme 1: Ineffectiveness and trial-and-error of treatments*

Many users report frustration with the unpredictability and inconsistency of pet treatments. Owners often mention trying multiple medications or methods without reliable results.

*1.3.2 Theme 2: Anxiety, pain, and emotional distress in pets*

A recurring theme involves treatments leading to or failing to alleviate anxiety, pain, or distress, especially in dogs. Owners describe how treatments are not addressing root causes and sometimes even exacerbate the discomfort.

*1.3.3 Theme 3: Concerns over over-medication and side effects*

Several posts mention concerns about overuse of medications, harsh side effects, and a preference for more natural or holistic approaches.

*1.3.4 Theme 4: Financial strain and accessibility issues*

Treatment costs are a major burden. Pet owners frequently reference the high expense of vet visits, medications, and emergency care, making them question the value or accessibility of some treatments.

*1.3.5 Theme 5: Rescue and foster pets with untreated conditions*

Many posts discuss pets (especially seniors or rescues) who arrive with long-neglected conditions and how ineffective or delayed treatments have had lasting impacts.

*1.3.6 Theme 6: Mistrust toward vets and pharmaceutical advice*

There is a strong undercurrent of mistrust toward veterinary advice, particularly when treatment seems profit-driven or when multiple vet opinions contradict each other.

*1.3.7 Theme 7: Emotional toll on pet owners*

Owners often describe the emotional burnout and sadness involved in managing chronic illness in their pets, especially when treatments are not effective. This is more prominent among those with long-term caregiving roles.

# Supplementary Tables

Facebook does not support Boolean search expressions therefore 32 separate Facebook searches were conducted. The Facebook search format is generally composed of list A terms and list B terms in combination, with a maximum of 20 search terms per Facebook data scrape. Each term from list A was combined with a term from list B, as shown in Table S1.

Table S1. Facebook search term lists. Each term from list A was combined with a term from list B and a total of 32 searches were run on Pulsar Platform™.

| **List A** | **List A cont.** | **List B** | **List B cont.** | **List B cont.** |
| --- | --- | --- | --- | --- |
| dog | poodle | pill | capsules | worming |
| dogs | rottweiler | pills | pilling | prescriptions |
| puppy | boxer | tablet | oral drugs | prescribed |
| pup | husky | tablets | medication | hide medicine |
| hound | doberman | by mouth | medications | in food |
| terrier | great dane | feed meds | chewables | in treats |
| labrador | saint bernard | feeding meds | flea chews | in cheese |
| golden retriever | mastiff | chewable | tick chews | in peanut butter |
| german shepherd | newfoundland | prescription | flea meds | in hamburger |
| bulldog | wolfhound | capsule | dewormer | in ham |
|  |  |  |  | oral medication |
|  |  |  |  | oral medications |

# Supplementary Figures


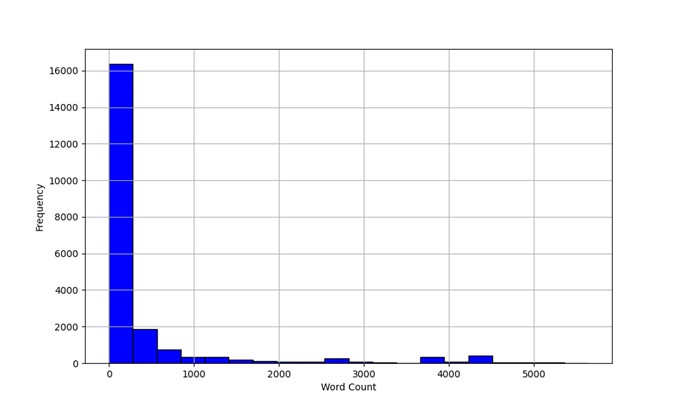


**Figure S1. Number of words per post versus the number of posts. This histogram demonstrates the justification for using the cut-off of >640 words to optimize the zero-shot relevancy filtering and to prepare the dataset for further analysis.**


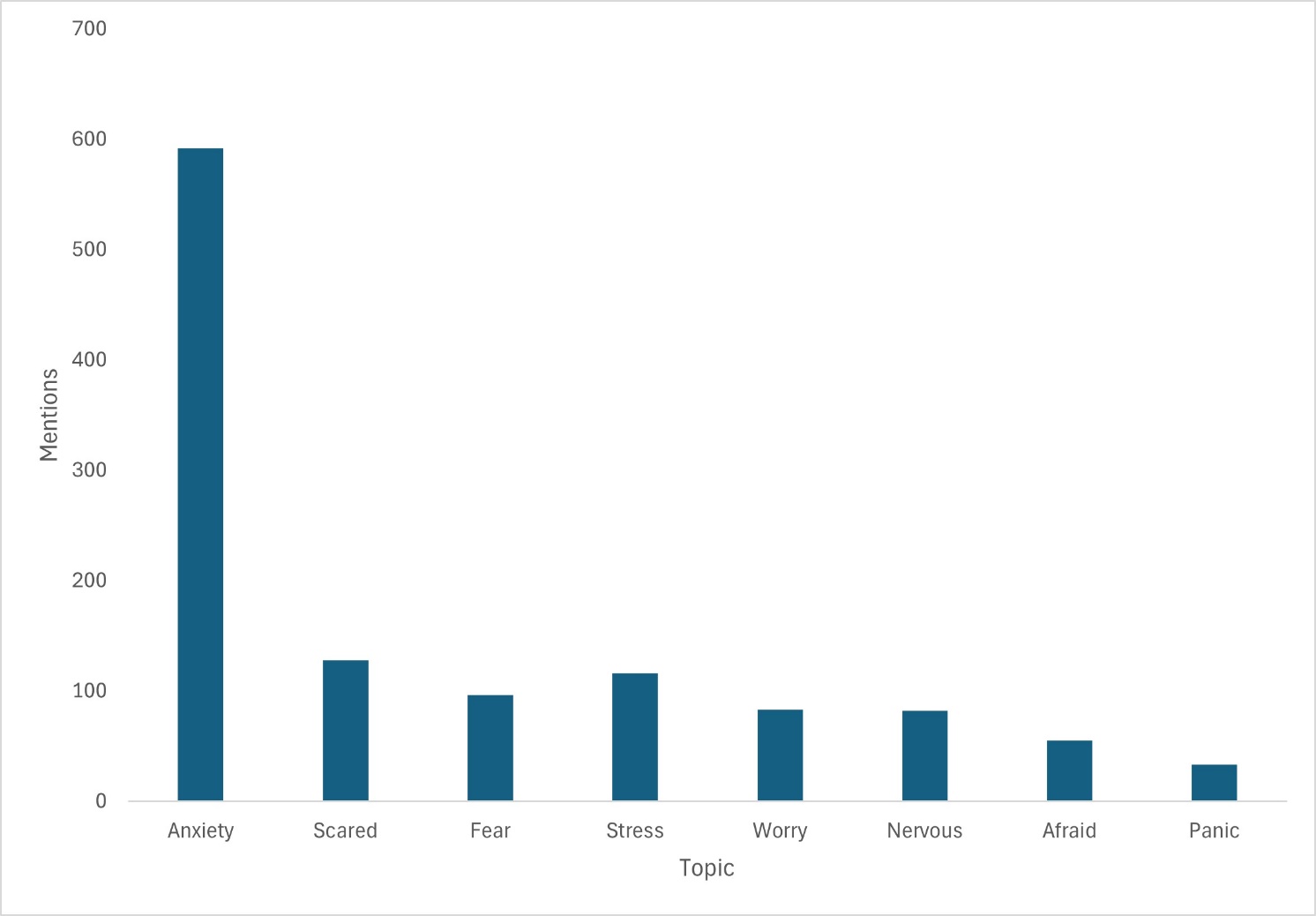


**Figure S2. Manual thematic analysis to find mentions of anxiety and related keywords in the text content of social media posts authored by pet owners.**
